# Posterior cingulate cross-hemispheric functional connectivity predicts the level of consciousness in traumatic brain injury

**DOI:** 10.1038/s41598-017-00392-5

**Published:** 2017-03-24

**Authors:** Haosu Zhang, Rui Dai, Pengmin Qin, Weijun Tang, Jin Hu, Xuchu Weng, Xing Wu, Ying Mao, Xuehai Wu, Georg Northoff

**Affiliations:** 1 Department of Neurosurgery, Huashan Hospital of Fudan University, NO. 12 Wulumuqi mid Road, Jing’an District Shanghai, 200040 China; 2 Center for Cognition and Brain Disorders (CCBD), Hangzhou Normal University. NO. 19#3 Shuyuan, NO. 58 Haishu Road, Yuhang district, Hangzhou, Zhejian Province 310000 China; 3 Institute of Mental Health Research, University of Ottawa, 1145 Carling Avenue, Room 6435, Ottawa, ON K1Z 7K4 Canada; 4 School of Life Science, South China Normal University, Key Laboratory of Ecology and Environmental Science in Higher Education of Guangdong Province, Guangzhou, 510631 PR China; 5 Guangdong Key Laboratory of Mental Health and Cognitive Science, South China Normal University, Guangzhou, 510631 China; 6 Centre for Studies of Psychological Applications, South China Normal University, Guangzhou, 510631 China; 7 School of Psychology, South China Normal University, Guangzhou, 510631 China

## Abstract

Previous studies have demonstrated that altered states of consciousness are related to changes in resting state activity in the default-mode network (DMN). Anatomically, the DMN can be divided into anterior and posterior regions. The anterior DMN includes the perigenual anterior cingulate cortex and other medial prefrontal cortical regions, whereas the posterior DMN includes regions such as the posterior cingulate cortex (PCC) and the temporal parietal junction (TPJ). Although differential roles have been attributed to the anterior and posterior DMN regions, their exact contributions to consciousness levels remain unclear. To investigate the specific role of the posterior DMN in consciousness levels, we investigated 20 healthy controls (7 females, mean age = 33.6 years old) and 20 traumatic brain injury (TBI) patients (5 females, mean age = 43 years old) whose brain lesions were mainly restricted to the bilateral frontal cortex but retained a well-preserved posterior DMN (e.g., the PCC and the TPJ) and who exhibited varying levels of consciousness. We investigated the intra- and cross-functional connectivity strengths (FCSs) between the right/left PCC and the right/left TPJ and their correlation with consciousness levels. Significant reductions in both the intra- and cross-hemispheric FCSs were observed in patients compared with controls. A significant correlation with consciousness levels was observed only for the cross-hemispheric PCC-TPJ FCS but not for the intra-hemispheric PCC-TPJ FCS. Taken together, our results show that the cross-hemispheric posterior DMN is related to consciousness levels in a specific group of patients without posterior structural lesions. We therefore propose that the PCC may be central in maintaining consciousness through its cross-hemispheric FC with the TPJ.

## Introduction

The default-mode network (DMN) is one of the central resting state networks in the brain^1^ and has been associated with various functions ranging from self-related processing^2–4^, mind wandering^5^, random thoughts, episodic memory retrieval and mental time travel^6, 7^. In addition, recent studies of individuals in altered states of consciousness, such as under anesthesia or in an unresponsive wakefulness/vegetative state (UWS/VS), have reported alterations in the resting state functional connectivity in the DMN^4, 8–10^. Despite several studies, the exact functional role of the DMN specifically regarding consciousness remains unclear.

Anatomically, the DMN can be divided into anterior and posterior regions. The anterior DMN includes the perigenual anterior cingulate cortex and other medial prefrontal cortical regions, whereas the posterior DMN includes regions such as the posterior cingulate cortex (PCC) and the temporal parietal junction (TPJ)^11, 12^. Although differential roles have been attributed to the anterior and posterior DMN regions^13^, their exact functions remain unclear. Studies of altered consciousness have observed changes in both the anterior and posterior DMN^14^ (see above). This finding raises questions regarding the exact and possibly distinct roles of the anterior and posterior DMN regions and their specific contributions to consciousness.

The posterior midline regions, such as the PCC, exhibit high degrees of resting metabolism^15^. Moreover, recent studies have suggested that the PCC may be central for consciousness^9, 16, 17^. We therefore hypothesized that the levels of hemispherical functional connectivity in the PCC may be related to consciousness levels.

The difference in the function between the left and right hemispheres has been previously discussed^18, 19^, but its relation to consciousness levels remains unknown. In addition, the specific effects posterior DMN, including the PCC, and their relation to the levels of consciousness are not well defined. Therefore, the current study sought to analyze both the intra- and cross-hemispheric functional connectivity between the PCC and the TPJ in a unique sample of patients with selective frontal lesions but no anatomical structural lesions in the posterior DMN and who exhibited varying levels of consciousness. Based on our own^9, 20^ and previous^12, 21–23^ results, we hypothesized that despite the absence of structural lesions the PCC-TPJ FCS may be reduced and related to the level of consciousness.

The aim of our study was to identify the impact of resting state functional connectivity in the posterior DMN (PCC, TPJ) on the level of consciousness in a sample of subjects without lesions in the posterior DMN. We investigated a sample of subjects with brain lesions primarily restricted to the bilateral prefrontal cortex but with well-preserved posterior DMN regions, e.g., the PCC and the TPJ, and varying levels of consciousness.

## Methods

### Subjects

The experiment included 20 healthy control subjects (7 females; 25–59 years old; mean age = 33.6 years) and 23 bilateral frontal injury cases (6 females; 22–63 years old, mean age = 43 years; 3 patients including 1 female and 2 males were excluded after preprocessing as they corresponded to less than 100 volumes). All human studies were approved by the Ethics Committee of Huashan Hospital and have therefore been performed in accordance with the ethical standards laid down in the 1964 Declaration of Helsinki and its later amendments. All patients provided their informed consent prior to inclusion in this study. These patients, who were enrolled when they were physically stable and did not received any consciousness-related drugs within one week, mainly suffered from bilateral frontal contusions with an intact posterior DMN (no visible traumatic or bleeding areas detected from MRI T1-weight, FLAIR and CT images, which were checked and rechecked by both neurosurgeons and radiologists). Table 1 provides the demographic and clinical data of the enrolled patients.

**Table 1:** Patient Information.

No	Sex	Age	Time^#^ (days)	Lesion Location	GCS^#^	CRS-R^#^	Lesion Type
1	M	47	98	Bilateral frontal lobes, temporal lobe	15	23	Traffic accident
2	M	50	20	Bilateral frontal lobes, temporal lobe	15	23	Traffic accident
3	M	60	217	Bilateral frontal lobes	14	23	Traffic accident
4	M	22	21	Bilateral frontal lobes	9	7	Traffic accident
5	M	40	104	Bilateral frontal lobes	12	20	Traffic accident
6	M	46	57	Bilateral frontal lobes	15	23	Tumble injury
7	M	18	15	Bilateral frontal lobes	9	14	Traffic accident
8	M	45	361	Bilateral frontal lobes	12	18	Traffic accident
9	M	37	83	Bilateral frontal lobes, temporal lobes	9	10	Traffic accident
10	M	45	39	Bilateral frontal lobes	9	13	Strike
11	M	42	31	Bilateral frontal and temporal lobes, bilateral basal ganglia	10	14	Strike
12	M	28	146	Bilateral frontal lobes, midbrain	7	5	Traffic accident
13	M	63	146	Bilateral frontal lobes	15	23	Traffic accident
14	M	34	138	Bilateral frontal lobes, brain stem	10	10	Strike
15	M	42	130	Bilateral frontal lobes	15	23	Tumble injury
16	F	29	10	Bilateral frontal lobes	9	8	Traffic accident
17	F	52	126	Bilateral frontal lobes	15	23	Strike
18	F	61	194	Bilateral frontal and temporal lobes, brain stem	7	6	Traffic accident
19	F	60	41	Bilateral frontal lobes	15	23	Traffic accident
20	F	56	116	Bilateral frontal lobes	15	23	Traffic accident

The mean interval between MR imaging and trauma = 109.25 days (range 20~361 days). GCS and CRS-R assessments were conducted immediately before radiological tests. P.S.: **Time** ^#^: The time from the day of injury to fMRI scanning day; **GCS** ^#^ **, CRS-R** ^#^ **:** Glasgow Coma Scale and Coma Recovery Scale-Revised assessment.

All subjects had no history of drug or alcoholic addiction, cerebrovascular or mental diseases, nor any congenital brain diseases (i.e., congenital hydrocephalus).

### Data acquisition

A Siemens Magnetom Verio 3.0 T MRI scanner (Siemens, German) with a standard head coil was used to scan the patients in the Department of Radiology of Huashan Hospital, Shanghai, China. The gradient-echo EPI images of the whole brain parameters were set as follows: 33 slices, repetition time/echo time [TR/TE] = 2,000/35 ms, slice thickness = 4 mm, field of view = 256 mm, flip angle = 90°, image matrix: 128 × 128). In total, 200 scans (406 seconds) were acquired in the resting run. In addition, high-resolution anatomical images were acquired for all subjects. T1-weighted magnetization-prepared rapid-gradient echo (MPRAGE) sequences were acquired through the sagittal plane with the following parameters: TR = 2300 ms; TE = 2.98 ms; TI = 900 ms; flip angle = 9°; matrix size = 256 × 240; slice number = 176; field-of-view (FOV) = 256 × 240 mm; acquisition averages = 1; scanning time = 301s. Patients’ CT scans were collected for detecting and identifying cerebral lesions. If the quality of the fMRI data were not satisfactory, such as being confounded by excessive head movements, a scan was performed again on a subsequent day.

All subjects’ MRI scans were acquired under resting states. For each patient, consciousness assessment were performed according to the standardized Glasgow Coma Scale (GCS) and CRS-R^24^.

### Data Analysis

All MR imaging data were analyzed using Analysis of Functional Neuroimage (AFNI) software (Cox, 1996; http://afni.nimh.nih.gov/afni). Due to the severe injury/lesions to the frontal lobes, spatial normalization was not performed, and the individual analysis was conducted. The preprocessing steps included head motion correction^25^, slice timing correction, spatially smoothing with a 6 mm full-width at half-minimum (FWHM) isotropic Gaussian kernel and temporal normalization. Scans with head motion greater than 0.5 mm of displacement^9^ in the x, y or z direction or 1.5 degrees of any angular motion throughout the course of the scan were excluded^26, 27^. Any subjects with less than 100 volumes were completely excluded. According to those standards, 3 patients (1 female and 2 males) were excluded from the experiment who corresponded to less than 100 volumes. The resting functional data were then filtered with a band-pass filter reserving signals between 0.01 and 0.1 Hz, which is regarded as the main reflection of neural fluctuations^28^.

After the head motion correction procedure, the magnitude of the head motion at each time point for 6 parameters (3 for shift and 3 for rotation) was obtained for each subject. The averaged head motion parameters for shift and rotation were then calculated via the method reported in the studies by Zang^29^ and Wu^17^ as follows:

$$\begin{array}{rcl}{\rm{Ms}} & = & \sum (|s{X}_{i}-s{X}_{i-1}|+|s{Y}_{i}-s{Y}_{i-1}|+|s{Z}_{i}-s{Z}_{i-1}|)/199\\ {\rm{Mr}} & = & \sum (|r{Y}_{i}-s{Y}_{i-1}|+|r{P}_{i}-r{P}_{i-1}|+|r{R}_{i}-r{R}_{i-1}|)/199\\ {\rm{Mt}} & = & {\rm{Mr}}+{\rm{Ms}}\end{array}$$

where Ms and Mr denote the averaged head motion parameter of shift and rotation, respectively, and Mt denotes the total head motion parameter; i denotes the time point of the time series ranging from 2–200; sX, sY and sZ denote the magnitude of the shift in the X, Y and Z directions, respectively; and rY, rP and rR denote the magnitude of yaw, pitch and roll rotations, respectively. Then, independent T tests were used to compare Ms, Mr and Mt between the patients and controls. There was no head motion difference between the controls and patients (*P*_Ms_ = 0.098 > 0.05; *P*_Mr_ = 0.440 > 0.05; *P*_Mt_ = 0.235 > 0.05).

**Figure 1:**
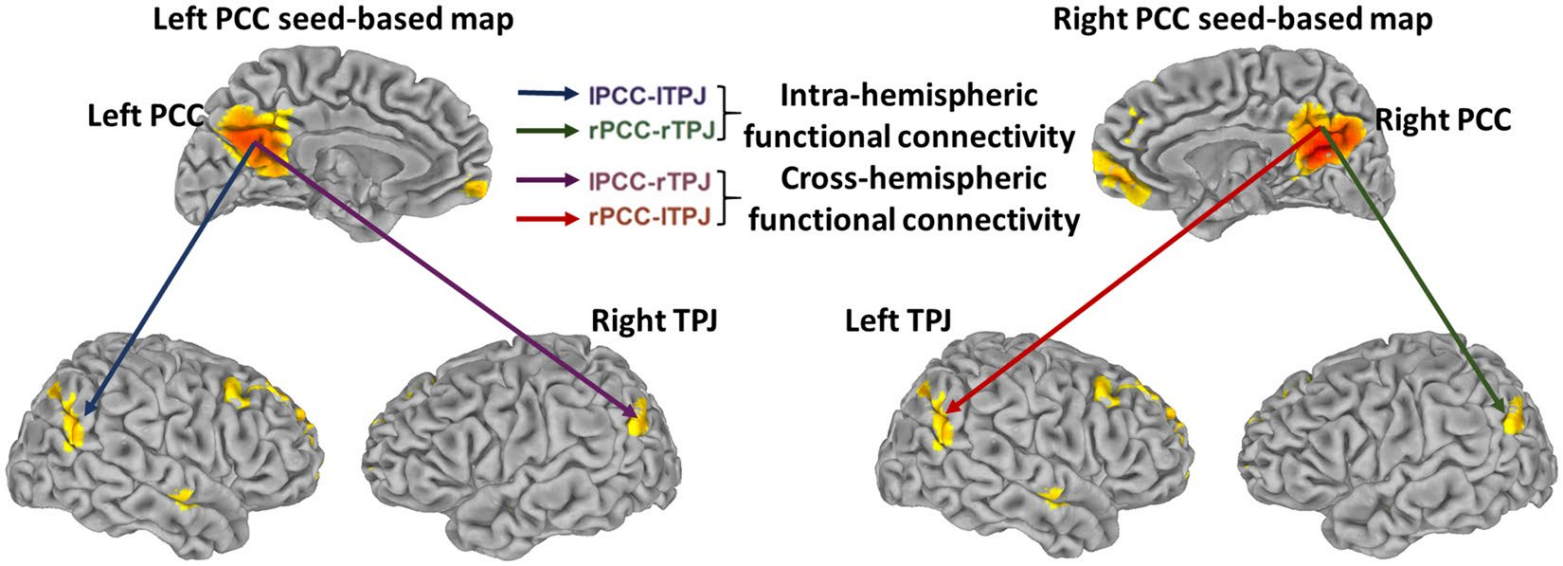
The images show one representative subject’s functional connectivity strength; the region of the PCC was used as the seed for analyzing the following FCSs: the FCS between the left PCC and left TPJ (intra-hemispheric FCS); FCS between the left PCC and right TPJ (cross-hemispheric FCS); FCS between the right PCC and left TPJ (cross-hemispheric FCS); the FCS between right PCC and right TPJ (intra-hemispheric FCS).

Then, during nuisance signal regression, 6 head motion-related regressors and the average signals from the CSF and white matter were regressed out^17, 30, 31^. After the PCC seed was chosen, the PCC seed-based functional connectivity map was acquired. Then, the correlation coefficients R of the PCC-TPJ (see the definition of TPJ below) for individual subjects were extracted and transformed to Fisher’s Z. The hemispheric Z scores, which represented the seed-based hemispheric FC strength indicating the relationships of lPCC-lTPJ and lPCC-rTPJ (from the left PCC seed-based map) and rPCC-lTPJ and rPCC-rTPJ (from the right PCC seed-based map) (see in Fig. 1) were extracted for further analysis. Here, ‘l’ and ‘r’ indicate that the partitions of the PCC and TPJ were in the left and right hemispheres, respectively. Individual anatomical segmentation via AFNI was conducted in the patient group, and the volumes of white matter and grey matter were recorded as covariate grey and white matter volumes for later partial correlation analysis. All data were imported into IBM SPSS (version 20) for statistical analysis (All the aforementioned results were thresholded at P < 0.05).

### Region Selection

To detect alterations in the posterior DMN, the PCC seed-based FC (functional connectivity) was used, as calculated by AFNI. The intra- and cross-hemispheric differences of the right/left PCC seed-based FCSs have not been previously investigated in frontal TBI patients.

As the incomplete brain structure, the left/right hemispheric PCC seeds were set individually for both the control and patient groups according to the following criteria: (1) the posterior cingulate cortex is the backmost part of the cingulate cortex, lying behind the anterior cingulate cortex. The PCC forms part of the posteromedial cortex with the retrosplenial cortex (Brodmann areas 29 and 30) and the precuneus (located posterior and superior to the PCC)^14^; (2) posterior to corpus callosum splenium at 0.5 cm; (3) perpendicular to the parietooccipital sulcus at 0.5 cm; (4) near the midline of the brain; (5) spheres with radii of 0.5 cm are drawn for the left PCC (lPCC) and right PCC (rPCC) seed regions.

After the PCC seed-based FC maps were calculated, we defined the TPJ ROI (Region of Interest) according to the FC map with the following criteria: (1) the anatomical temporoparietal junction lies in the region between the temporal and parietal lobes near the Sylvian fissure. Specifically, it is composed of the inferior parietal lobule and the caudal parts of the superior temporal sulcus; (2) Also, according to the hemispherical PCC based functional connectivity map, the highest correlation cluster (within anatomical TPJ) was included in TPJ ROI; (3) spheres with radii of 0.5 cm are drawn in hemispherical TPJ regions as the left TPJ (lTPJ) and right TPJ (rTPJ) ROIs, respectively. All values shown for the functional connectivity in all the posterior regions are ROI-based rather than voxel-based. All of the determined ROIs were double checked by the second (R.D.) and second to last author (X.W.) of the paper.

### Statistical analysis

Between- and intra-group multiple comparisons of the intra- and cross-hemispheric PCC-TPJ FCSs from patients and healthy subjects were performed. Then, an intra- and between-group repeated-measures ANOVA was conducted to detect differences among the PCC-TPJ FCS patterns of the healthy and patient groups. To investigate the relationship between the PCC-TPJ FCS and the level of consciousness, we conducted two types of analyses. First, the patient group was divided into three subgroups for the levels of consciousness according to the high (GCS = 12–15), medium (GCS = 9–11), and low (GCS = 3–8) GCS scores. Due to the insufficient number of patients in the low group, the low and medium consciousness groups were combined (n = 9) for comparison with the high consciousness group (n = 11) using an independent sample t-test. Second, we conducted Spearman’s correlation analyses between the PCC-TPJ FCSs and the GCS scores and between the PCC-TPJ FCSs and the CRS-R scores. A partial correlation was conducted with covariates, including head motion (Mt) and grey and white matter volumes. All the aforementioned results were thresholded at P < 0.05.

## Results

A common way to detect the DMN and consciousness alterations of TBI patients is using the whole brain functional map, which may include the trauma regions and their peripheral areas. Previous studies have shown that these regions are located within anatomically damaged areas that contain astrocytes undergoing proliferation and repair^32, 33^; therefore, it is difficult to determine whether the origins of their signals are from conserved neurons. In patients with frontal lobe injuries, the reserved posterior regions may more accurately reflect changes in the DMN within DOC (Disorder of Consciousness) states.

### Comparison of different PCC-TPJ resting state functional connectivity patterns

**Figure 2:**
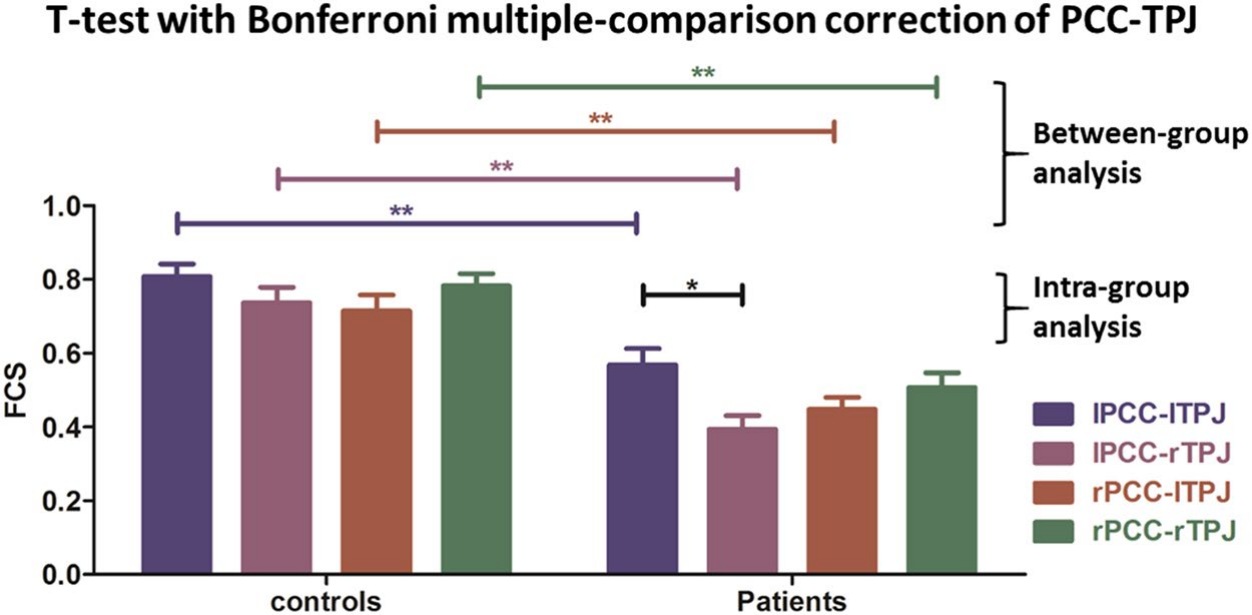
The bars represent the relative magnitudes of the FCSs (mean+/− SE). The general linear model (univariate) test showed that there were differences between the controls and patients (F = 99.626; P < 0.01), and the T-test with Bonferroni multiple-comparison correction demonstrated that (1) for the between-group analysis, both the intra- and cross-hemispherical FCSs of the patients decreased compared with controls; (2) for the intra-group analysis, a difference was detected between the left intra-hemispheric (lPCC-lTPJ) FCS and the cross-hemispheric (lPCC-rTPJ) FCS (F = 3.094; P = 0.011, corrected) for the patient group, whereas no significant difference among the PCC-TPJ patterns was observed in the control group (F = 1.215; P = 0.310, corrected). **α = 0.01; *α = 0.05.

An analysis of the FCSs between ROIs was performed (rather than voxel-based; see above). A 2-way repeated-measures mixed ANOVA with pairs of regions as within-group factor and a between-group factor was conducted and revealed differences between the controls and patients (F = 99.626; P < 0.01), significant differences among 4 types of PCC-TPJ patterns (F = 3.920; P = 0.010 < 0.05), and no significant interaction effect of the PCC-TPJ patterns and the subject groups (F = 0.639; P = 0.591 > 0.05). Then, the T-test with Bonferroni multiple-comparison correction was used to detect differences among all of the PCC-TPJ FCS patterns between controls and patients. This test revealed significant decreases in both the intra- and cross-hemispheric resting state FCSs of the patient group (See Table 2 and Fig. 2). An intra-group one-way repeated measures ANOVA with within-group factor analysis of the controls showed that there was no significant difference among the PCC-TPJ FCS patterns (F = 1.215; P = 0.310 > 0.05). However, in the patient group, the intra-group general linear model (univariate) revealed differences among the PCC-TPJ FCS patterns (F = 3.099; P = 0.011 < 0.05), significant differences among the levels of consciousness (F = 19.090, p < 0.01), and no significant interaction effect of the level of consciousness and the PCC-TPJ patterns (F = 0.763; P = 0.519 > 0.05, corrected). Then, the T-test with Bonferroni multiple-comparison correction was used to detect differences between the left intra-hemispheric (lPCC-lTPJ) and the cross-hemispheric (lPCC-rTPJ) FCSs (F = 3.094; P = 0.011 < 0.05, corrected) (See Fig. 2).

**Table 2:** Significant T-test with Bonferroni multiple-comparison correction Results of PCC-TPJ Patterns between Patients and Controls.

Items		Difference of mean	P
Controls	Patients		
lPCC-lTPJ	lPCC-lTPJ	0.242	0.001**
lPCC-rTPJ	lPCC-rTPJ	0.353	0.000**
rPCC-lTPJ	rPCC-lTPJ	0.274	0.000**
rPCC-rTPJ	rPCC-rTPJ	0.290	0.000**

T-test with Bonferroni multiple-comparison correction: SE = 0.058. **α = 0.01; *α = 0.05.

### Relationship between intra- and cross-hemispheric PCC-TPJ resting state functional connectivity and consciousness levels in patients

**Figure 3:**
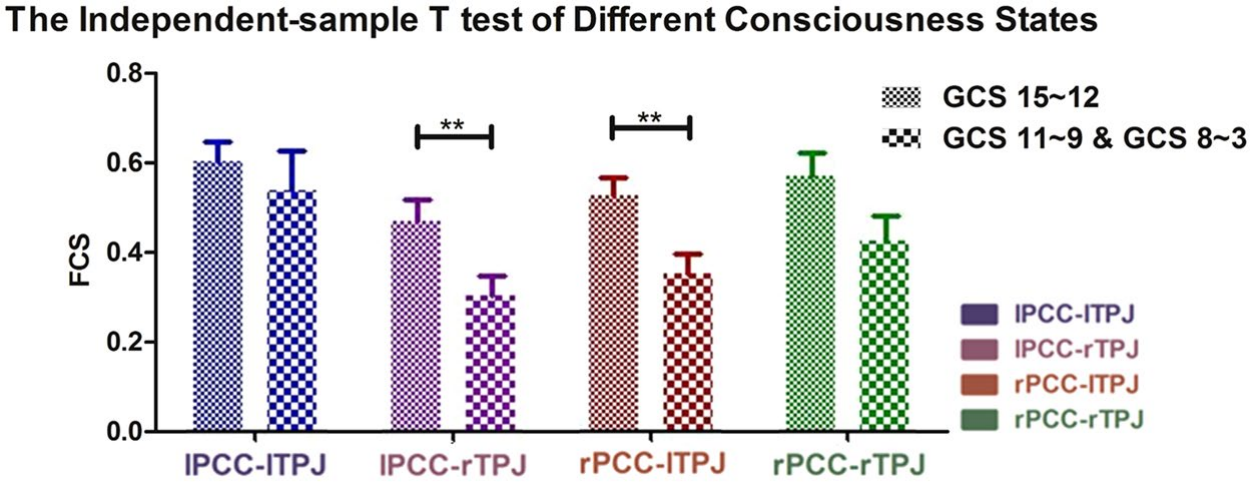
Differences in the PCC-TPJ FCSs between the low/medium and high GCS subgroups of patients (low/medium GCS: 9 patients, high GCS: 11 patients) using an independent group analysis. The bars represent the relative magnitudes of the FCSs (mean+/− SE). **Low/medium GCS versus high GCS:** lPCC-lTPJ (F = 3.932; P = 0.126); lPCC-rTPJ (F = 0.607; P = 0.002 < 0.01); rPCC-lTPJ (F = 0.008; P = 0.004 < 0.01); rPCC-rTPJ (F = 0.072; P = 0.315). The cross-hemispherical FCSs represented the major difference between the low/medium GCS and high GCS groups. **.α = 0.01; *.α = 0.05.

First, independent t-tests were conducted in 2 subgroups of patients according to the GCS scores: high-scoring (GCS = 12–15) patients (n = 11) were compared with a subgroup of medium- (GCS = 9–11) and low- (GCS = 3–8) scoring patients (n = 9). This result revealed significantly lower PCC-TPJ FCSs in the medium/low group than in the high group. A highly significant difference was found for the cross-hemispheric PCC-TPJ FCS between the two subgroups, whereas a significant difference was not detected in the intra-hemispheric PCC-TPJ FCS (see Fig. 3).

**Figure 4:**
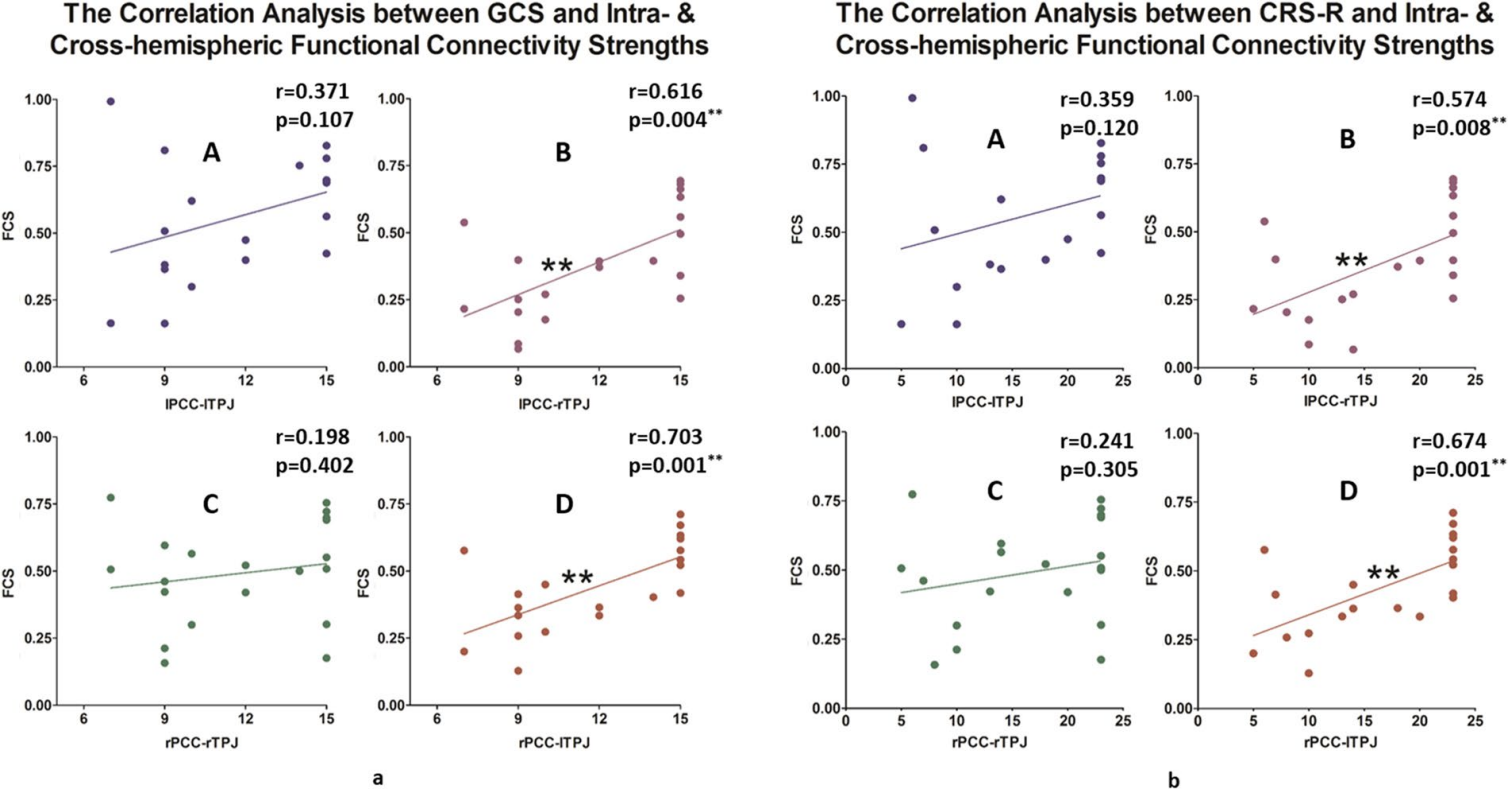
(**A**,**B**) The Spearman’s correlation of the PCC-PTJ FCSs with both the GCS scores and the CRS-R scores in the TBI patient group. The cross-hemispherical FCSs were significantly correlated with the consciousness level for both scales. (**A,C**): Intra-hemispheric functional connectivity (lPCC-lTPJ and rPCC-rTPJ). (**B,D**): Cross-hemispheric functional connectivity (lPCC-rTPJ and rPCC-lTPJ). **α = 0.01; *α = 0.05.

Second, Spearman’s correlation analyses were conducted between the PCC-TPJ resting state FCS and the GCS scores and between the PCC-TPJ resting state FCS and the CRS-R scores (See Table 3 and Fig. 4). Interestingly, only the cross-hemispheric PCC-TPJ resting state functional connectivity predicted the level of consciousness (see Table 3 and Fig. 4A,B). Neither the CRS-R scores nor the GCS scores were correlated with the intra-hemispheric PCC-TPJ FCS.

**Table 3:** Correlation Analysis of the Intra- and Cross-hemispheric Functional Connectivity Strengths with both the GCS^#^ and CRS-R^#^ scores.

Items		GCS^#^	CRS-R^#^
lPCC-lTPJ	correlation coefficient	0.371	0.359
	*P*	0.107	0.120
lPCC-rTPJ	correlation coefficient	0.616	0.574
	*P*	0.004^**^	0.008^**^
rPCC-lTPJ	correlation coefficient	0.703	0.674
	*P*	0.001^**^	0.001^**^
rPCC-rTPJ	correlation coefficient	0.198	0.241
	*P*	0.402	0.305

This table shows the statistical data corresponding to Fig. 4A,B. P.S.: **GCS** ^#^ **:** Glasgow Coma Scale assessment. **CRS-R** ^#^ **:** CRS-Revised assessment. **α = 0.01; *α = 0.05.

**Table 4:** Partial Correlation Analysis of the Intra- and Cross-hemispheric Functional Connectivity Strengths with both the GCS and CRS-R Scores.

Covariate	Items		GCS^#^	CRS-R^#^
Head motion, grey and white matter volumes	lPCC-lTPJ	correlation coefficient	0.227	0.222
		*P*	0.266	0.376
	lPCC-rTPJ	correlation coefficient	0.611	0.528
		*P*	0.007^**^	0.024^*^
	rPCC-lTPJ	correlation coefficient	0.579	0.550
		*P*	0.012^*^	0.018^*^
	rPCC-rTPJ	correlation coefficient	0.332	0.355
		*P*	0.179	0.149

This table shows the partial correlation statistical data between the PCC-TPJ FCSs and the consciousness assessments (GCS and CRS-R) with covariates of head motion (Mt) and grey and white matter volumes. P.S.: **GCS** ^#^ **:** Glasgow Coma Scale assessment. **CRS-R** ^#^ **:** CRS-Revised assessment. **α = 0.01; *α = 0.05.

Finally, to exclude effects of brain volumes and head motion, a partial correlation analysis with covariates (head motion data (Mt), grey and white matter volumes) were conducted between the PCC-TPJ resting state FCS and the GCS scores (Table 4). As before, only the cross-hemispheric PCC-TPJ FCS was positively correlated with consciousness levels (whereas no correlation was obtained for the intra-hemispheric PCC-TPJ FCS). In regard to the CRS-R scores, we observed the same pattern of correlation.

## Discussion

In the current study, we investigated the intra- and cross-hemispheric resting state functional connectivities in the posterior DMN in a unique sample of bilateral frontal-injured patients with varying levels of consciousness and with no underlying anatomo-structural lesions in the posterior DMN. The main findings are as follows: (i) significantly decreased resting state PCC-TPJ FCS was observed in the frontal lesion patients compared with the healthy subjects; (ii) there was a difference between the left intra- and cross-hemispheric FCSs in the patient group; and (iii) a significant correlation between the cross-hemispheric PCC-TPJ FCSs and the level of consciousness was observed, whereas such a correlation was not observed for the intra-hemispheric PCC-TPJ FCSs. By enrolling a unique group of TBI patients with no posterior DMN lesion, our study showed for the first time the functional impact of the posterior DMN on the level of consciousness with a special role in the cross-hemispheric PCC-TPJ resting state functional connectivity.

We therefore suggest that cross-hemispheric FCS (as distinguished from intra-hemispheric FCS) in the posterior DMN regions between the PCC and the TPJ may play a particularly central role in contributing to the level of consciousness.

In our study, the results demonstrated that the posterior DMN could be affected by frontal lesions and reflect the levels of consciousness, which is in line with previous studies^34, 35^. Our study extends upon these previous results by showing cross-hemispheric PCC-TPJ changes in a special group of patients with selective frontal lesions but without structural posterior DMN lesions. While controlling for various covariates (head motion, grey matter and white matter volumes), significant correlations were detected between the cross-hemispheric resting state PCC-TPJ functional connectivity and the level of consciousness including a higher cross-hemispherical FCS (lPCC-rTPJ and rPCC-lTPJ) and a higher level of consciousness. By contrast, we did not find any such correlation between the intra-hemispherical FCS and the level of consciousness.

The relation of the PCC-TPJ FCS with the MPFC and the relation between the anterior and posterior regions of the DMN remain unclear. All our patients showed bilateral MPFC lesions; therefore, we were unable to subgroup our patient sample according to different degrees of MPFC lesions. Future studies should therefore test the impact of the MPFC on PCC-TPJ, such as during anesthesia, when results would not be confounded by lesions to either the MPFC or the PCC.

The right and left TPJ are related to different functions, such as self-produced actions and attention in the right TPJ and language cognition, processing, and comprehension of both written and spoken language in the left TPJ^36^. Our results extend these observations by showing a specific role of cross-hemispheric PCC-TPJ FCS in the level of consciousness: the PCC’s communication with the TPJ in the contralateral hemisphere seems to be central for maintaining the level of consciousness. This finding is even more remarkable given that both the intra- and cross-hemispheric resting state PCC-TPJ FCSs were reduced in the TBI group, in accordance with the whole-brain FCS decrease observed in DOC patients in previous studies^37, 38^. This result suggests a specific role for cross-hemispheric PCC-TPJ resting state functional connectivity in maintaining (or deteriorating) and reflecting the level of consciousness.

The PCC is a core region of the posterior DMN located in both hemispheres, which has been shown to be related to consciousness in previous studies^35, 39^. These studies showed PCC signal changes in DOC patients conducting auditory tasks^9^, a link between temporal variability and signal synchronizations that include PCC-region signals during wakefulness that is disrupted during anesthesia^40^, and decreases in the PCC signals in subjects undergoing hypnosis^41^. Our results are consistent with these results and extend them with regard to a specific role of cross-hemispheric FC of the PCC to the TPJ. Moreover, because our subjects exhibited exclusive frontal lesions without lesions to posterior DMN regions, the cross-hemispheric PCC-TPJ effects must be considered functional rather than based on structural lesions.

However, the exact origin of the disrupted PCC-TPF FCS remains unclear. First, it may be due to micro-lesions in the PCC hemispheric connecting fibers. Second, the origin may also be related to disturbed functional connectivity of the PCC and the TPJ to the anterior DMN regions, such as the MPFC regions^42^, which have been shown to be associated with consciousness^35, 43, 44^. Due to their anterior lesions, the cingulate bundle, which passes from anterior to posterior DMN regions, may be affected. This notion requires future investigations via methods such as diffusion tensor imaging (DTI). However, our results clearly demonstrate the importance of cross-hemispheric interactions and cross-hemispheric functional connectivity^38^ in TBI patients and in subjects with altered states of consciousness in general.

In conclusion, we investigated a unique TBI patient group with varying levels of consciousness and absent anatomo-structural lesions in the posterior DMN regions. This made our group of subjects particularly suitable for investigating the role of the posterior DMN in consciousness. We observed significant decreases in both intra- and cross-hemispheric PCC-TPJ resting state functional connectivity in the TBI patient group compared with the healthy subjects. However, only cross-hemispheric (rPCC-lTPJ and lPCC-rTPJ) resting state FCSs (but not intra-hemispheric resting state FCSs) predicted the level of consciousness in the TBI patient group. This result suggests a special, although yet unclear, role of cross-hemispheric posterior DMN resting state FCS in maintaining consciousness.

## Electronic supplementary material

Author contribution statement
