# Supplementary material for: Posterior cingulate cross-hemispheric functional connectivity predicts the level of consciousness in traumatic brain injury
Source: Sci Rep. 2017 Mar 24;7:387. doi: 10.1038/s41598-017-00392-5 (PMC5428308; doi:10.1038/s41598-017-00392-5)
Supplement: Supplementary file 1 — Author contribution statement [file 41598_2017_392_MOESM1_ESM.doc]

**Posterior cingulate cross-hemispheric functional connectivity related to consciousness in traumatic brain injury**

**Author contributions statement:**

**Haosu Zhang:** Project development, Image analysis, Statistic calculation, Manuscript writing

**Xuehai Wu**: Project development, Image analysis, experimental plan establishment, Funds and Project owner, Post author.

**Georg Northoff**: Project development, experimental plan establishment, Funds owner, Post author.

**Ying Mao:** Project development, experimental plan establishment

**Rui Dai:** Image analysis, Manuscript writing, co-author

**Pengmin Qin**: Project advisor, Manuscript correction.

**Weijun Tang:** Data collection, MRI scanner operator, Anatomical location analysis

**Jin Hu:** Data collection

**Xuchu Weng:** Project advisor

**Xing Wu:** Data collection
